# The Regulation of Fat Metabolism during Aerobic Exercise

**DOI:** 10.3390/biom10121699

**Published:** 2020-12-21

**Authors:** Antonella Muscella, Erika Stefàno, Paola Lunetti, Loredana Capobianco, Santo Marsigliante

**Affiliations:** Department of Biological and Environmental Science and Technologies (Di.S.Te.B.A.), University of Salento, 73100 Lecce, Italy; erika.stefano@unisalento.it (E.S.); loredana.capobianco@unisalento.it (L.C.); santo.marsigliante@unisalento.it (S.M.)

**Keywords:** lipid metabolism, endurance exercise, plasma fatty acids, lipoprotein, high-density lipoprotein (HDL), low-density lipoprotein (LDL)

## Abstract

Since the lipid profile is altered by physical activity, the study of lipid metabolism is a remarkable element in understanding if and how physical activity affects the health of both professional athletes and sedentary subjects. Although not fully defined, it has become clear that resistance exercise uses fat as an energy source. The fatty acid oxidation rate is the result of the following processes: (a) triglycerides lipolysis, most abundant in fat adipocytes and intramuscular triacylglycerol (IMTG) stores, (b) fatty acid transport from blood plasma to muscle sarcoplasm, (c) availability and hydrolysis rate of intramuscular triglycerides, and (d) transport of fatty acids through the mitochondrial membrane. In this review, we report some studies concerning the relationship between exercise and the aforementioned processes also in light of hormonal controls and molecular regulations within fat and skeletal muscle cells.

## 1. Introduction

Regular physical activity is important not only for mental health but also for physical health. Exercise training has implications in epigenetic regulation [1], aging [2], improvement of glycemic control in patients with type 2 diabetes mellitus and insulin sensitivity and resistance [3,4], prevention of cardiovascular diseases [5,6,7], and others such as multiple sclerosis, lung diseases, Parkinson’s disease, and so on [8,9,10,11,12,13]. Thus, the study of lipid metabolism is a key element to understand how physical activity influences our health and, in particular, that of professional athletes. Several studies have highlighted the differences between athletes and sedentary subjects, although some differences among sport disciplines exist [14].

Fat and carbohydrate provide the most important form of fuel for exercise and sports activities. During exercise, there are four major endogenous sources of energy: plasma glucose derived from liver glycogenolysis, free fatty acids (FFAs) released from adipose tissue lipolysis and from the hydrolysis of triacylglycerol (TG) in very low-density lipoproteins (VLDL-TG), and muscle glycogen and intramyocellular triacylglycerols (IMTGs) available within the skeletal muscle fibers. Fats and carbohydrates are oxidized simultaneously, but their relative contribution depends on a variety of factors, exercise duration and intensity included. Substrate utilization as fuel sources during physical activity is also highly influenced by the type of exercise.

Endogenous triacylglycerols represent the largest energy reserve in the body, 60 times greater than the amount of energy stored as glycogen. In a lean adult man, most triacylglycerols are stored in adipose tissue (≈17,500 mmol), skeletal muscle (≈300 mmol), and plasma (≈0.5 mmol) [15]. Furthermore, liver and pancreas, together with muscle, represent ectopic fat deposition sites [16]. Actually, the amount of FFA available from muscle triglycerides is not accurately known; in fact, it is not easy to discriminate between FFA coming from the lipid droplets inside the muscle fibers or from the adipocytes present between the fibers [17,18]. However, the significant quantity of FFA used during exercise comes solely from adipose tissue and muscle [17,19,20]. The substrate used to derive energy during exercise depends on the duration and intensity of the latter: glucose utilization is greater during high exercise intensity, while FAs oxidation increases during moderate exercise intensity [21,22]. In fact, there is a balance between carbohydrate and lipids that influence their utilization: this phenomenon is called ‘Randle cycle’ and consists of inhibiting glucose uptake and oxidation in muscle when FAs oxidation is intense. Conversely, ‘reverse Randle cycle’ occurs when hyperglycemia can reduce FAs oxidation [23].

This review mainly concentrates on findings in humans, and particular attention will be paid to lipid metabolism during aerobic exercise, particularly emphasizing hormonal controls and molecular regulations within fat and skeletal muscle cells. It is acceptable to imagine that understanding how and why lipid metabolism varies during physical performance can improve health through aerobic exercise. Articles included in the review are shown in Table 1.

## 2. Lipids as a Form of Energy during Exercise

Fat digestion occurs in the duodenum, due to the pancreatic lipase that releases monoacylglycerols (MAG), diacylglycerols (DAG), and FFA. Long-chain FA (LCFA) are absorbed into duodenum and reformed in triglycerides which, together with cholesterol and proteins, constitute the chylomicrons and are transported into the blood. Muscle and fat cells receive FAs from chylomicrons [24]. Triglycerides and plasma cholesterol are transported in four main classes of lipoproteins: (1) chylomicrons, (2) very low-density lipoproteins (VLDL) rich in triglycerides, (2) intermediate-density lipoproteins (IDL), (3) low-density lipoproteins (LDL) rich in cholesterol, and (4) high-density lipoproteins (HDL). 

High-density lipoprotein plays an essential role in plasma lipid transport, providing to the metabolism of chylomicrons and VLDL and acting as a scavenger of surplus unesterified cholesterol from these lipoproteins. The chylomicron particle number remains unchanged following acute and chronic aerobic exercise [25,26]. Interestingly, after six months of resistance exercise intervention in diabetic adults, a significant reduction in the concentration of apo B48 is obtained, which is present in chylomicrons and in their remnants [27]. In addition, resistance exercise decreases triglycerides and cholesterol within chylomicrons, in healthy sedentary men [28], and also endogenous and meal-derived FA incorporation into chylomicron-TG and TRL-TG, in overweight/obese men with prediabetes [29]. The aerobic and/or resistance exercise decrease total cholesterol and LDL-C and increase high-density lipoprotein- cholesterol (HDL-C) [30,31]. High-density lipoprotein- cholesterol concentrations are inversely associated with risk for cardiovascular disease [32], thus exercise interventions are routinely prescribed to decrease the risk of cardio-metabolic complications by promoting an increase in HDL-C concentration [31,33,34,35]. Unfortunately, recent clinical trials aimed at reducing the risk of cardiovascular disease by increasing HDL-C levels have been unsuccessful [36]. However, several studies also showed that exercise training caused changes in HDL subclasses, favoring increases in larger HDL subclasses, independent of changes in body composition [31,37,38]. In addition, in adults with CVD, diabetes mellitus, and metabolic syndrome, regular exercise has beneficial effects on various HDL functions, including endothelial protection [39], antioxidative [38,40], and anti-inflammatory properties [41,42]. Although VLDLs represent the main source of circulating triglycerides both in fasting and fed states, the FAs from labeled VLDL-TG were shown to comprise 3% of total energy utilization [43] or 13% of total FA oxidation [44] during moderate-intensity exercise in humans. VLDLs are converted to lipoproteins with intermediate (IDL) and low (LDL) densities, having low levels of triglycerides [19,45,46,47,48]. During fasting, FAs provide both local energy and ketone bodies that represent an energy source for heart and kidneys but are not regarded as part of the true triglyceride energy pool.

### 2.1. Fat Metabolism and Endurance Training

The contribution of carbohydrate and fats to the body’s energy production depends on exercise duration and intensity, training condition, sex, body composition, and diet [49]. Since, at rest, the FAs released from adipose tissue surpass the quantity of FAs oxidized in the skeletal muscles, most of the FAs are re-esterified into liver triglycerides [15]. Then, fats are mainly oxidized at rest and at low aerobic exercise intensities, while carbohydrates are chiefly used at high intensities of exercise.

#### 2.1.1. The rate of Lipolysis is Modulated by Temperature

Environmental temperature may also have some effects. Environmental heat stress increases muscle glycogenolysis, hepatic glucose output, and whole-body carbohydrate oxidation rates, whilst it decreases fat oxidation rates at given intensities. From this, it could lead to the hypothesis that maximal fat oxidation (MFO) decreases in the heat compared to temperate conditions [50,51]. However, more recently, O’Hearn et al. [52] rated FFA concentration and oxidation in eight male subjects after passive heating at 42 °C for 120 min and following exercise on a treadmill in the same temperature at 50% VO_2max_ for 30 min. Plasma FFA concentration was significantly higher both following passive heating and exercise, compared to the control group (exercise at 23 °C), whereas TG, cholesterol, and phospholipid levels did not differ. The high FFA concentration in the passively heated group was not related to a whole-body FA oxidation [52]. The effect of cold environments on substrate metabolism during prolonged exercise is less certain. Some investigations have reported augmented carbohydrate utilization in cold vs. temperate conditions [53,54], whereas others suggested that fat utilization is augmented, and carbohydrate utilization is suppressed in the cold. The data disparities are probably due to interactions between the specific environmental conditions and exercise modality (cycling vs. running) [55]. For example, during moderate-intensity cycling, greater fat oxidation rates at 11 °C than at 21 °C were reported, but this was suppressed at 4 °C [53]. When carbohydrate and lipid oxidation were examined in six males rested for 3 h at 29 °C and at 5 °C, it was observed that cold increased plasma glucose and plasma FFA ratios. In spite of enhanced lipolysis, only about half the rate of FFA is ultimately oxidized [56]. However, exercise performance could be influenced by several factors that must be taken into account. For example, the surrounding medium (air or water), the exercise intensity, individual’s anthropometric characteristics, body composition, and clothes can influence results obtained at the same temperature [57].

#### 2.1.2. The Rate of Lipolysis Is Modulated by the Intensity of Physical Activities

The metabolism of lipid includes lipolysis, their transport in the blood to the cytosol of the muscle, and the FAs transport to the mitochondria of running muscles to be oxidized in order to produce a great quantity of ATP. In the following sections, some studies regarding the relation between the mentioned phases and endurance training and physical fitness are reported. Articles included in the review are shown in Table 1.

Consistent with its central importance in lipid and energy homeostasis, lipolysis occurs in essentially all tissues and cell types. FAs derived from adipose tissue, muscle lipid droplets, and diet represent the main energy supply during exercise with intensities between 45% and 65% VO_2max_ [58]. At a low to moderate intensity, as well as during prolonged exercise, most of the energy requirements for skeletal muscle can be met from predominantly FA oxidation, with a small contribution from glucose oxidation. On the contrary, glucose predominates as an energy substrate during short-term intense exercise [59]. Thus, when exercise intensity increases, the use of fat to total oxidative metabolism decreases [60,61]. The index that establishes the training load is the maximal oxygen consumption (also named as maximal oxygen uptake or maximal aerobic capacity, VO_2max_), which is the maximum amount of oxygen that can be used in the unit of time by an individual, during a physical activity. VO_2max_ varies over a wide range among individuals, depending on level of aerobic training, genetic makeup, age, health status, and sex. It defines functional aerobic capacity of a single individual in a specific exercise performance and reflects a person’s cardiorespiratory fitness level [62].

The source of FA changes during exercise: at 25% of VO_2max_, the oxidized fat derives from plasma FAs [20,60,61,63,64]. When exercise intensity increases, there is a shift from FA to glucose oxidation with a reduction in the percentage of the total energy requirement derived from fat oxidation and a reciprocal increase in carbohydrate oxidation, which becomes the main energy source when exercise reaches above ~80% of VO_2max_ [58,65,66,67]. During exercise intensity at 65% of VO_2max_, the contribution of plasma FAs decreases and the rate of IMTG increases and provides about 50% of the FA for total fat oxidation [20,68,69]. Thus, peripheral lipolysis and, consequently, the release rate of FFA into plasma, is stimulated at maximum at the lowest exercise intensity and progressively decreases with increasing exercise intensity up to a point where the concentration of plasma FFA during exercise at 85% of VO_2max_ appears significantly suppressed.

Fatty acids uptake from plasma lipoprotein triacylglycerols represents less than 3% of the energy consumed during prolonged exercise [67,83]. The increase of lipolysis and, consequently, the release rate of FFA in the plasma, is greater in endurance-trained, with respect to untrained subjects [63]. The majority of the studies have shown a decrement of TG after aerobic exercises, due to their mobilization from visceral and sub-cutaneous adipose tissues along with TG in the VLDL-C broken down to FFA by lipases [68,69,80,81]. It is well-known that marathon and middle-distance runners have different protocols of endurance training. While middle-distance runners adopt fast and discontinuous exercises, marathon runners execute most continuous running exercises. Then, the middle-distance runners, being faster, have a higher anaerobic capacity than marathon runners who have a higher VO_2max_, keeping in mind that different training histories and genetic differences exist [84].

Muscle TG lipolysis is stimulated by high-intensity exercises. Therefore, after a high-intensity exercise, while lipolysis is immediately decreased, the release of FFA into the plasma increases, indicating that these derive from previously hydrolyzed triglycerides during the recovery [20]. Also, the marathon performance level correlates to a decrease of blood TG and to a proportional glycerol concentration increase, as revealed in a study performed on 14 top-class marathon runners, after a 10 km run at their individual marathon velocity [69]. In addition, in marathon runners, a significant glucose concentration increment, a longer and/or less unsaturated blood FA, and a higher aminoacidic production and blood release (resulting by catabolism of several proteins for amino acid supply to skeletal muscle), were also reported [69]. These results show that both carbohydrate, lipid, and amino acid metabolisms are necessary to improve energetic supply to skeletal muscle during runner exercise. Thus, the lipolytic response should not be different between endurance-trained and untrained men. In fact, plasma glycerol and FFA rate of appearance raised similar values in both five endurance-trained (with 4 h of treadmill exercise) and five control subjects [63]. A considerable blood FAs increment, during and after the race, was also measured in 18 non-professional, middle-aged runners of a 2-day ultramarathon (130 km). Conversely, plasma TG decreased on days 2 and 3, while HDL-C was elevated from day 2 to day 5 [81]. After moderate-intensity endurance exercise, lipolysis remains significantly elevated compared to rest for up to 24 h, thus even a single bout of exercise can influence energy expenditure/balance over the next day [85].

Hetlelid et al. [86] demonstrated a three times higher fat oxidation in elite runners compared to non-elite runners during high-intensity exercise. Aslankeser and Balc [87] observed 17 times higher fat oxidation in an athlete group compared to an untrained group during high-intensity intermittent exercise (80% VO_2max_), while carbohydrate oxidation rate was the same in trained and untrained subjects.

In 2017, Nieman et al. [72] conducted a study on twenty-four male runners in order to evaluate changes in metabolic profile related to exercise intensity of 70% VO_2max_. After running, an increase in FA oxidation products (dicarboxylate and monohydroxy fatty acids, acylcarnitine) and ketone bodies as well as a decrease in muscle glycogen was found [70].

In 15 runners (age, 35.2 ± 8.7 years) undergoing three days of intensified training, a severe systemic change in blood metabolites related to energy production, especially from the lipid metabolism, was observed. They ran for 2.5 h/day on treadmills at 70% VO_2max_ for three days in a row, and immediately after the exercise period, a significant increment in 22 metabolites related to lipid/carnitine metabolism was measured, which was not fully restored to pre-exercise levels, not even after 14 h recovery [73]. Such intensified exercise provoked an increase in biomarkers related to carnitine, long-chain FAs, dicarboxylate, and essential FA metabolisms, and decreases the metabolites related to lysolipid and bile acid metabolism. Finally, the pattern of change in key metabolites did not differ between genders [73].

As stated above, plasma lipids and carbohydrates are used at the onset of exercise and during moderate exercise intensity, while intracellular stored substrates are needed during high exercise intensity. Nonetheless, IMTG are used in lower quantities than plasma FFA and they cannot compensate for the reduction in plasma FFA oxidation. Therefore, IMTG are not an energy source that is rapidly usable, and muscle glycogen utilization is necessary during continuous moderate exercise [74,88]. However, during exercise, TG oxidation is greater in the trained than in the untrained subjects. But, during recovery, plasma glycerol and FFA decrease more rapidly in trained than in control subjects. Thus, during low-intensity exercise, endurance runners utilize more fat than sedentary healthy men do [63].

Total fat oxidation and the rate of appearance of glycerol and FFA during exercise at 40% VO_2max_ was assessed in five volunteer cyclists [73]. During endurance exercise, the increase in fat oxidation was principally due to the decrease of re-esterification; with the start of recovery, however, the percent re-esterification rose to 90%, and during the first 20 min of recovery, lipolysis rapidly decreased but it resulted still significantly elevated after 2 h of recovery [70].

After 12 weeks of endurance training, plasma fatty acid oxidation decreases [89], suggesting an increased dependence on IMTGs as a fuel of energy [15]. Since training does not alter abdominal or femoral adipose tissue lipolysis, the palmitate rate of appearance in plasma and plasma FA oxidation, the extra FA utilized during exercise possibly comes from non-plasma stores. Therefore, fat metabolism in response to endurance training is localized to IMTG stores. Specifically, the depletion of IMTG of type I muscle fibers is higher than that of type II muscle fibers, since type I fibers contain approximately twice the IMTG of type II fibers [61,90]. Furthermore, the different types of muscle fibers influence FA oxidation capacity during exercise not only for the greater amount of IMTG, but also because of the lipolytic and oxidative enzymes. Thus, endurance-trained subjects have a higher maximal fat oxidation rate since they have more type I fibers that express high adipose triglyceride lipase (ATGL), hormone-sensitive lipase (HSL), Perilipin-5 (PLIN5), 3-Hydroxyacyl-CoA dehydrogenase (HAD) and Oxidative phosphorylation (OXPHOS) complexes levels [75].

The greater use of fat, as an adaptive response to endurance sports, is related to mitochondrial quantitative and qualitative adaptations: increment of skeletal muscle mitochondrial volume density and intrinsic mitochondrial fatty acid oxidation [15,91]. Both adjustments have been reported in a study performed on eight competitive male cross-country skiers compared to eight untrained controls [76]. It was seen that the mitochondrial volumetric density, mitochondrial fatty acid oxidation, VO_2max_, and maximal fat oxidation (at 46% VO_2max_) were higher in endurance athletes than in controls. In addition, maximum fat oxidation and mitochondrial volume density were correlated in endurance athletes, suggesting that mitochondrial volume expansion and density could limit speed for maximum fat oxidation [76].

Finally, fat oxidation increases from rest to low- and moderate-intensity exercise (maximum at about 60–65% VO_2max_) but decreases at power outputs above approximately 75% VO_2max_. Increasing the exercise intensity above approximately 50% VO_2max_ also increases the use of muscle glycogen, while carbohydrate oxidation increases during exercise at higher, compared with moderate, exercise power outputs.

### 2.2. Regulation of FAs Oxidation in Skeletal Muscle during Exercise

#### 2.2.1. Exercise Intensities’ Effects on Beta Oxidation

Hetlelid et al. [86] demonstrated a three times higher fat oxidation in elite runners compared to non-elite runners during high-intensity exercise. Aslankeser and Balc [87] observed 17 times higher fat oxidation in an athlete group compared to an untrained group during high-intensity intermittent exercise (80% VO_2max_), while carbohydrate oxidation rate was the same in trained and untrained subjects.

In 2017, Nieman et al. [72] conducted a study on twenty-four male runners in order to evaluate changes in metabolic profile related to exercise intensity of 70% VO_2max_. After running, an increase in FA oxidation products (dicarboxylate and monohydroxy fatty acids, acylcarnitine) and ketone bodies as well as a decrease in muscle glycogen was found [70].

In 15 runners (age, 35.2 ± 8.7 years) undergoing three days of intensified training, a severe systemic change in blood metabolites related to energy production, especially from the lipid metabolism, was observed. They ran for 2.5 h/day on treadmills at 70% VO_2max_ for three days in a row, and immediately after the exercise period, a significant increment in 22 metabolites related to lipid/carnitine metabolism was measured, which was not fully restored to pre-exercise levels, not even after 14 h recovery [73]. Such intensified exercise provoked an increase in biomarkers related to carnitine, long-chain FAs, dicarboxylate and essential FA metabolisms, and decreases the metabolites related to lysolipid and bile acid metabolism. Finally, the pattern of change in key metabolites did not differ between genders [73].

#### 2.2.2. Molecular Mechanisms Regulating FAs Oxidation

Regulation of FAs oxidation in skeletal muscle during exercise is due to a series of tightly coordinated molecular events. Among these molecular mechanisms, there are evidences demonstrating that acetyl-CoA availability in the mitochondrial matrix adjusts FAs oxidation to exercise intensity and duration. The rate of glycolysis seems to be central to mitochondrial acetyl-CoA availability and hence the regulation of FAs oxidation. During high-intensity aerobic exercise, glycolytic flux is increased and enhanced pyruvate production leads to acetyl-CoA excess, which in turn is buffered through catalase (CAT) enzyme. This discharges pyruvate dehydrogenase complex (PDH) inhibition, enabling increased glucose oxidation to sustain ATP resynthesis [92]. In fact, an important step in the subsequent absorption of FAs into the mitochondria is their conversion into fatty acyl-CoA esters, allowing to keep FAs within the cell and to establish a gradient. This process is controlled by acyl-CoA synthetase (ACS), which converts FAs into acyl-CoA by-products. Skeletal muscle cells possess several ACS isoforms with different subcellular localization and affinities for FAs; between these, ACSL1 isoform is important for FAs oxidation during exercise in skeletal muscle [59].

**Figure 1:**
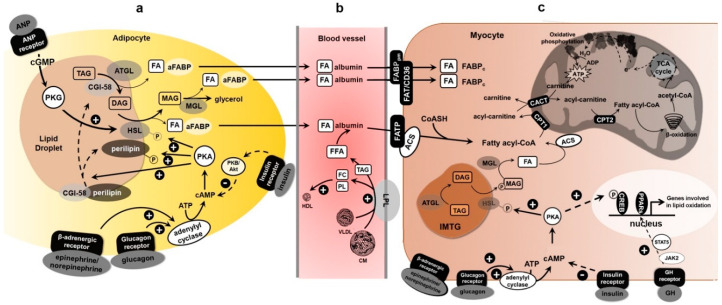
Fatty acid mobilization and utilization in skeletal muscle during endurance exercise. (**a**) Epinephrine (as well as norepinephrine) and glucagon stimulate FA release from TG stored in adipocyte lipid droplets, with insulin countering their actions. Epinephrine and glucagon bind their specific receptor in the adipocyte membrane, thus stimulating adenylyl cyclase to produce cyclic AMP (cAMP). cAMP activates the cAMP-dependent protein kinase (PKA), which phosphorylates both HSL and perilipin present on the surface of the lipid droplet. The phosphorylation of perilipin increases ATGL activity, thereby providing more diacylglycerol (DAG) substrates to hormone-sensitive lipase (HSL). Hormone-sensitive lipase then hydrolyzes DAG to a free fatty acid (FFA) and MAG, which is further hydrolyzed by a monoacylglycerol lipase (MGL). FFAs are transported to the plasma membrane bound to adipocyte fatty acid-binding protein (aFABP), leave the adipocyte, and bind serum albumin in the blood. (**b**) Exercise induces lipoprotein lipase (LPL) on the surface of endothelial cells of skeletal muscle. The increased LPL activity increases TG hydrolysis from TG-rich lipoproteins (such as chylomicrons (CM) and very-low density lipoproteins (VLDL)), thus releasing FFA, glycerol, free cholesterol (FC) and phospholipids (PL). The esterified cholesterol is packaged into the core of HDL particles, increasing plasma HDL-C levels. (**c**) FFA derived from lipoproteins and adipocyte lipolysis are released from the albumin and enter myocytes via specific fatty acid transporters, such as fatty acid translocase (FAT/CD36), plasma membrane-associated fatty acid binding proteins (FABP_pm_) and fatty acid transport proteins (FATP). Long-chain FAs bind directly to FATP closely associated with sarcolemmal acyl-Coenzyme A synthethase (ACS). Alternatively, FAs may first bind to FAT/CD36 and then be delivered either to FATP or to cytosolic fatty acid binding proteins (FABP) and activated into acyl-Coenzyme A (acyl-CoA) by intracellular ACS. Acyl-CoA esters enter the mitochondrion via carnitine palmitoyl transferase 1 (CPT1) and are cleaved in the β-oxidation pathway. The acetyl-Coenzyme A molecules produced are oxidized through the tricarboxylic acid cycle (TCA), and the energy of oxidation is conserved in ATP, which fuels muscle contraction and other energy requiring metabolism in the myocyte. FA released from intramyocellular triacylglycerol store (IMTG) through local HSL activity also contribute to lipid utilization in the myofibers during this exercise type.

Long-chain FAs, which constitute the majority of the FFAs obtained in the diet or released from adipose tissue, unlike short- and medium-chain FAs, cannot pass directly through the mitochondrial membranes but, due to the carnitine palmitoyl transferase 1 (CPT1), they are transformed into fatty acyl carnitine derivatives (Figure 1c). CPT-1, located at the outer mitochondrial membrane, exists in two isoforms: liver-type (L-CPT1) and muscle-type (M-CPT1). In skeletal muscle, the M-CPT1 isoform is predominant [93].

Acyl carnitine is transported through the mitochondrial inner membrane in exchange for a free carnitine molecule by a translocase (CACT) and is reconverted to acyl-CoA by CPT2 inside the mitochondrion for oxidation [94] (Figure 1c). During exercise at increasing intensity, a parallel increase in muscle acetyl-CoA and acetylcarnitine content by one- to three-fold compared with rest or low-intensity exercise is found; correspondingly, the free carnitine content fell from 75% of the total muscle carnitine at rest to 20% at an exercise intensity of 90–100% of VO_2max_. These results suggest that acetylcarnitine is a major metabolite formed during intense muscular effort and that carnitine has a function in the regulation of the acetyl-CoA/CoA ratio by buffering excess production of acetyl units [95,96,97].

Results also suggest that carnitine availability per se is a key regulator of muscle fuel selection, inasmuch as an increase in skeletal muscle total carnitine content provokes the inhibition of carbohydrate oxidation in conditions of high carbohydrate availability. The decrease in muscle free carnitine availability when increasing exercise intensity, restricts CPT1 flux and consequently leads to a decrement in muscle long-chain fatty acid oxidation [61,98].

During intense exercise, when the rate of carbohydrate oxidation becomes maximal, an increase of mitochondrial piruvato deidrogenasi (PDH) activation is observed [99,100]. Conversely, the increase in muscle carnitine content in young, healthy volunteers modulated changes in whole-body energy expenditure, quadriceps muscle fuel metabolism and gene expression, due to the increase in muscle fat oxidation owing to increased muscle long-chain FAs translocation via CPT1 [101,102,103]. Therefore, carnitine, that stimulates the transport of long-chain FAs across the inner membrane of the mitochondrion and short-chain FAs across several membranes, may lead to a detoxification process, eliminating those metabolites that could damage organelles [104,105]. Regarding the benefits of oral administration of L-carnitine, studies are contradictory, showing no gain [106,107] or less chemical damage and muscle soreness [108,109,110,111] or a better and faster recovery [112]. Since oxidation of the medium-chain FA octanoate is unchanged when exercise intensity shifts from 40% to 80% of VO_2max_, medium-chain FAs should be able to bypass CPT1, as was the case for oleate, a CPT1-dependent long-chain FA [113].

Exercise induces FA transporter (FAT/CD36) translocation from intracellular stores to the mitochondrial membrane in muscle [114,115], where it interacts with ACSs regulating fatty acyl-CoA availability to CPT1 [116], thus suggesting a regulatory role of FAT/CD36 in mitochondrial FA oxidation during exercise. Fat oxidation can also be limited by high concentrations of malonyl-CoA, the product of the acetyl-CoA carboxylase (ACC) reaction, an allosteric regulator of CPT1 activity in vitro [117]. There are two ACC isoforms, ACC1 (or ACCα) and ACC2 (or ACCβ), expressed in several tissues and in skeletal muscle respectively, according to hormonal responses and nutritional status [118,119]. In skeletal muscle, ACCβ activity and malonyl-CoA concentration decrease during increasing exercise intensity, leading to the increment of FA oxidation [120,121], sometimes also simultaneously to an increase of 5’ AMP-activated protein kinase (AMPK) activity [122]. Conversely, inactivation of ACC results in a decrease in malonyl-CoA, thus reducing CPT1 inhibition and contributing to FA oxidation increase from rest to exercise [123]. Therefore, the modulation of ACC and CPT-1 activities can influence the quantity of intramuscular FAs that are oxidized as an energy source relative to their stored amount as triacylglycerol.

During submaximal exercise, with a bicycle ergometer for 70 min (10 min at 40% and 60 min at 65% VO_2max_), FA oxidation increases, and skeletal muscle malonyl-CoA content remains unchanged [124]. Conversely, after 1 min exercise with a bicycle ergometer at 35% VO_2max_, malonyl-CoA content decreases but returns to rest level in 10 min and it does not decrease during cycling at 65% VO_2max_. Thus, a decrease in malonyl-CoA content is not required during FFA uptake and oxidation increment that occurs over exercise at 35 and 65% VO_2max_. Furthermore, since malonyl-CoA content does not change during exercise at 90% VO_2max_, it does not contribute to the lower rate of fat oxidation at this exercise intensity [125]. On the other hand, during one-legged knee-extensor exercise at 60%, 85% and 100% of VO_2max_, ACC activity decreases by 50–75%, accordingly, to exercise intensity, and such decrement is due to phosphorylation on Ser^221^ of ACCβ by AMPK [98].

The increase in mitochondrial enzymes of FA oxidation after endurance training also suggests a regulatory pathway of candidate genes activated by training [15]. PPARs (peroxisome proliferator-activated receptors) are a class of ligand-dependent nuclear transcription factors that are important for metabolic homeostasis. Whole-body lipolytic activity, plasma FA oxidation and PPARα amount in skeletal muscle, were measured in six lean women before and after 12 weeks of endurance training. In addition to a 25% increase in total FA oxidation during 90 min of bicycling exercise (50% pretraining peak O_2_ consumption), the training also increases of about two-fold the levels of muscle PPARα and, consequently, of its target proteins regulating FA oxidation (medium-chain and very long-chain acyl-CoA dehydrogenase (MCAD and VLCAD)) [15]. Studies performed on laboratory rodents showed that PPARβ is required in skeletal muscles for the maintenance of slow oxidative fibers and that ablation of PPARβ in skeletal muscles leads to obesity and diabetes [126]. Exercise increases skeletal muscle PPARδ expression in humans and rodents [127,128,129,130] and PPARδ represses glycolytic genes in muscle to slow glucose consumption in mouse [131]. Finally, the PPARδ agonist GW501516 has been classified as a doping substance by the World Anti-Doping Agency (WADA) due to its capability to influence gene expression [132].

EB transcription factor (TFEB) has been shown to translocate to nuclei during exercise and to induce mitochondrial biogenesis; accordingly, mice lacking muscle TFEB exhibit lower FA oxidation during exercise [133]. Finally, AMPK, which is involved in the regulation of both myocellular energy homeostasis and mitochondrial biogenesis, has also been proposed as a regulator of FA oxidation during exercise [134].

Thus, the regulation of FA oxidation in skeletal muscle during exercise is not due to a single mechanism or signaling pathway but is due to a set of closely coordinated molecular events depending upon metabolic fluxes.

## 3. Transport of FAs from Adipose Tissue to Skeletal Muscle and Exercise

The FAs released in the blood are transported bound to albumin [19,135] (Figure 1b). In order for FAs to enter the muscle, they must pass through the vasal endothelium, the interstitial space and then the sarcolemma of the muscle cell. On the endothelium, the FA-albumin complex interacts with specific albumin binding proteins (ABP), thus facilitating FA absorption by skeletal muscle. The increase in uptake of plasma FAs into skeletal muscle during exercise is governed by several highly coordinated and regulated transports: transmembrane, cytosolic and mitochondrial membrane, as well as intramitochondrial FA oxidation.

Despite the fact that FAs can easily enter and diffuse within biological membranes, there is now clear evidence that trans-sarcolemmal FA transport involves membrane-associated FA binding proteins (Figure 1c). The FA binding protein of the plasma membrane (FABPpm), fatty acid transport protein (FATP) and FAT/CD36 facilitate the passage of FA through the membrane. In the cytosol, FAs bind to the cytoplasmic FA-binding protein (FABPc); then, FAs are targeted to the mitochondria for oxidation or remain in the cytosol for re-esterification [136]. The expression of FA binding proteins differs according to cell types and their physiological function in the various districts has yet to be fully understood [137].

Fatty acid transport /CD36 is located on sarcolemma membrane and in endosome: exercise can induce its reversible translocation from sarcolemma to plasma and mitochondria membrane in order to facilitate FA transmembrane diffusion [59,138]. Muscle contraction increases FAT/CD36 protein content in the plasma membrane and reduces its content in intracellular membranes [139]. Moreover, humans deficient in FAT/CD36 decrease aerobic exercise capacity due to less FA uptake in muscle [82,140]. Manio et al. [137] demonstrated that FAT/CD36 is essential for basal endurance performance and improvement induced by training in mice. It is involved in PPAR-related transcriptional responses in muscle; in fact, FAT/CD36 KO mice have an inefficient upregulation of PPAR and PPAR-related exercise-responsive genes after training [137]. Conversely, high levels of FAT/CD36 enhance lipid oxidation during exercise [138].

It has been proven that high-intensity training increases FA transport protein contents in skeletal muscle [77]. Regarding FABPpm, its training-induced upregulation, in vastus lateralis muscle, is related to gender as changes are not obtained in women [141]. Interestingly, gender differences in FABPpm protein content are not seen in non-trained subjects [141,142]. Conversely, FAT/CD36 protein content is higher in females than in males [139]. Talanian et al. [77] observed an increase in both FABPpm and FAT/CD36 in ten untrained females. After 6 weeks of training, a larger increase of FAT/CD36 is found in whole muscle (10%) and mitochondrial membrane (51%), but not in sarcolemmal membrane. FABPpm content increased in total muscle (48%) and sarcolemmal membrane (23%), but not in mitochondria. FAT/CD36 protein content in vastus lateralis muscle is not different in female and male subjects [141] and short-term training for 9 days [143] or a single exercise bout [144] increase FAT/CD36 protein content by 20–25% in muscle. In addition, Bradley et al. [145] demonstrated that endurance cycling exercise at 60% VO_2max_ induces an increase in plasma membrane FAT/CD36 and FABPpm content in human skeletal muscle. AMPK may increase the translocation of FAT/CD36 and FABPpm [145,146], although the oxidation of FAs does not decrease in the absence of AMPK activity [147,148,149]. Jeppesen et al. [150] observed that lack of liver kinase B1 protein (LKB1), the primary kinase that mediates AMPK phosphorylation [151], drastically decreases FA oxidation in mice both during in vivo exercise and during contraction in isolated muscle ex vivo, suggesting that LKB1 is significant for FA oxidation in muscle during exercise, independently of AMPK [150].

FATPs are associated to FA uptake and oxidation (Figure 1b); in particular, FATP1 and FATP4 convert LCFAs to acyl-CoA thioesters and are mostly expressed in type I muscle fibers [152]. FATP1 seems to be associated to an increased lipid oxidation during prolonged submaximal exercise (45–80% VO_2max_). Indeed, in Jeppesen et al.’s study, FATP1 increases by 33% in skeletal muscle, while FATP4 decreases by 20% after 8 week of exercise training [78]. Jain et al. [153] found that both insulin and muscle contraction in mice stimulate the translocation of several FA transporters, such as FAT/CD36, FABPpm, FATP1 and FATP4, but not FATP6.

A single high-intensity exercise attack in males was reported to reveal exercise-regulated phosphorylation sites on 562 proteins [154], which underlines the extent of exercise-regulated kinases that can be potential candidates in the exercise-induced regulation of the FAT/CD36 translocation. It is also worth noting that other post-translational modifications of FAT/CD36, including ubiquitination, glycosylation, palmitoylation and acetylation [155], could exert regulatory effects on FAT/CD36 trafficking.

During muscle contraction and exercise, signaling pathways such as calcium/calmodulin-dependent protein kinase kinase (CaMKK) [156], extracellular regulated kinases 1/2 (ERK1/2) [140] and p38 mitogen-activated protein kinase [157] are activated, some of which have been linked to translocation of FAT/CD36 to the plasma membrane.

## 4. Molecular Regulation of Lipolysis and Exercise

In terms of molecular regulation, the main lipases activated during lipolysis are hormone-sensitive lipase (HSL), adipose triglyceride lipase (ATGL) and monoacylglycerol lipase (MGL) [16] (Figure 1a). The existing literature provides limited and contradictory data on adipose expression of ATGL and/or HSL during exercise. Petridou et al. [79] found that obese men had lower mRNA levels of ATGL and HSL compared with lean men, and in both groups no changes in mRNA levels were found during exercise. Instead, prolonged moderate-intensity exercise activated ATGL to a similar degree in subcutaneous adipose tissue of lean and obese young men and the patterns of activation were transient in the lean and prolonged in the obese. These results suggested posttranslational modifications, and the reversible ATGL and HSL phosphorylation seem to be the most probable candidate.

Hormone-sensitive lipase protein or mRNA has been detected in human skeletal muscle, but with a considerably lower expression than in adipose tissue [98]. The HSL protein expression also varies between fiber types, being higher in oxidative than glycolytic fibers [158]. HSL activity, both in adipose tissue and skeletal muscle, is regulated via phosphorylation–dephosphorylation, but also by allosteric mechanisms [159,160]. Five phosphorylation sites on HSL have so far been identified as regulatory sites. In vitro studies have demonstrated that Ser^563^, Ser^659^ and Ser^660^ are cAMP-dependent protein kinase A (PKA) targets on HSL. In adipocytes, all three sites are phosphorylated both in vivo with isoprenaline and in vitro when incubated with PKA [161,162].

### 4.1. Endocrine Regulation

During endurance exercise, the release of FFA and glycerol from the adipose tissue triglycerides stores into the plasma is stimulated by several lipolytic hormones, including glucagon [163], catecholamines (epinephrine and norepinephrine) [164,165], growth hormone (GH), atrial natriuretic peptide (ANP), brain natriuretic peptide (BNP) and cortisol [164,165,166]. Catecholamines regulate lipolysis through different adrenoceptors subtypes (β1, β2, β3, α1, α2) linked to stimulatory (G_s_) or inhibitory (G_i_) G-proteins able to stimulate or inhibit adenylate cyclase (AC), respectively. The major endocrine mechanism is epinephrine increase. Epinephrine, acting through PKA, activates ATGL to stimulate lipolysis and maintain non-esterified fatty acids supply during exercise, a response that is abolished following the blockade of β-adrenoreceptors with propanalol [167]. Human adipose tissue presents all β-adrenoceptors, with β1 and β2 being the most active in this tissue. Unlike the release of FFA from adipose tissue, muscle triglycerides are uniquely controlled by epinephrine through the β2-adrenoceptors [168,169]. β-adrenoreceptors are coupled to G_s_-protein, thus activating PKA and leading to the phosphorylation of HSL and perilipin 1. HSL/perilipin 1 complex translocates to the lipid droplets. Phosphorylated perilipin 1 releases comparative gene identification-58 (CGI-58), a protein currently known to regulate lipolytic enzymes directly and independently of cellular context [170] (Figure 1a). In fact, CGI-58 binds to ATGL, inducing lipolysis. α2-adrenoceptors are coupled to G_i_-proteins that leads to a reduction of PKA activation with a consequent inhibition of lipolysis [16,171]. However, Verboven at al. [71] observed that lipolysis in subcutaneous adipose tissue is mainly mediated by non-adrenergic factors in obese insulin-sensitive, obese insulin-resistant and lean insulin-sensitive men, whilst catecholamine-mediated lipolysis was reduced in obese insulin-resistant compared to insulin-sensitive subjects. In addition, some studies show lower exercise-induced lipolysis in obesity [79,172], which has been attributed to lower HSL gene expression [173,174].

Atrial natriuretic peptide (ANP) released from the heart acts in conjunction with the sympathetic nervous system in order to provide energy (lipid mobilization from adipose tissue) in stressed situations such as exercise [175]. Adenosine monophosphate-activated protein kinase (AMPK) is activated by ANP and in general, when lipolysis is induced in adipose tissue, exercise included [176,177,178]. 5’-AMP-activated protein kinase phosphorylation on Thr^172^ is increased in the subcutaneous adipose tissue of individuals exercising for 90 min at 60% VO_2max_ together with an increased plasma FA concentration [179]. Conversely, Kristensen et al. [180] found no change in AMPK activity after exercise of similar intensity for a shorter period (40 to 60 min) and for which plasma FA concentrations were not reported. Many studies suggest that, in human adipocytes, AMPK activation is a key process for maintaining energy homeostasis when lipolysis is activated. Atrial natriuretic peptide stimulates lipolysis through the activation of the type A guanylyl cyclase receptor (atrial natriuretic peptide receptor, NPR-A), bringing cyclic-GMP enhancement and activation of HSL [181]. During exercise, glucagon and cortisol also increase provoking ATGL activation through PKA [167] (Figure 1a). Also, high growth hormone (GH) levels induce lipolytic stimulation during prolonged fasting [182]. In adipocytes, GH levels are related to low fat-specific protein 27 (FSP27) expression that regulates lipolysis through the interaction with ATGL [183,184]. Otherwise, insulin inhibits lipolysis, but suppression of insulin secretion is not sufficient to increase it [58]. Chakrabarti et al. [185] demonstrated that insulin inhibits lipolysis and promotes triglyceride storage by decreasing ATGL gene expression. Furthermore, while plasma insulin decreases during exercise, GH increases after exercise, particularly during recovery, and with less extent in obese subjects [184]. Thus, aerobic endurance-trained athletes but also obesity phenotype influence plasma glucose oxidation and FFA levels. Indeed, a decrement in plasma glucose oxidation and FFA levels was shown during high and low exercise intensity, but with different kinetics in athletic or obese subjects [22].

### 4.2. Hormone-Sensitive Lipase (HSL) Functions

In vitro, fatty acyl-CoA and oleic acid inhibits HSL activity, whereby TG lipolysis decreases [186]. Fatty acyl-CoA has the greatest effect, decreasing HSL activity by about 50% [187]. During exercise, the muscular acyl-CoA content increases. HSL activity and fatty acyl-CoA content increase in skeletal muscle after 10 min of cycling at 60% of peak O_2_ uptake, increase further by 60 min and decrease to near-resting values by 120 min. HSL activity increment at 60 min is due to the stimulating effect of increased epinephrine and decreased insulin levels, whilst HSL activity decrement is associated with the inhibitory effects of the accumulation of acyl-CoA [188]. The allosteric inhibition of TG hydrolysis in muscle during exercise at high intensity, when intracellular accumulation of FAs occurs, could, in some cases, nullify the activation by phosphorylation. In addition, HSL phosphorylation might change its sensitivity towards fatty acyl-CoA, its allosteric regulator [189]. In the study by Watt et al. [190], the ingestion of nicotinic acid decreased the intramuscular fatty acyl-CoA concentration, thereby relieving the allosteric inhibition and conferring increased in vivo HSL activity; conversely, nicotinic acid decreased lipolysis in adipose tissue, supporting the knowledge that lipolysis is regulated differently in these two tissues [186]. Intramyocellular triacylglycerols lipolysis has been found in skeletal muscle in response to epinephrine, exercise and during the contractions of isolated muscles [144,191,192]. Furthermore, in skeletal muscle, TG lipase activity is increased both by epinephrine and by local factors in response to contractions [193,194]. In skeletal muscle, HSL is responsible for 20–60% of TG hydrolase activity during resting conditions [144,193,194,195,196], but HSL is considered the primary lipase activated by contractions and epinephrine [144,193,194,195]. However, studies on HSL-deficient mice revealed that these animals accumulated DAG rather than TG in adipose, muscle and testis tissues in response to fasting [197,198]. Thus, these studies suggest that TG lipases other than HSL may exist. Many studies demonstrate an exercise-induced increase in HSL activity in skeletal muscle [98,188]. Watt et al. [188] showed that HSL activity increases when measured in male subjects at three different exercise training intensities (30%, 60% and 90% of VO_2max_) and such greater HSL activity does not differ between exercise intensities. Furthermore, HSL activation increases in untrained subjects from rest to exercise at 70% of VO_2max_ and remains unchanged when increasing exercise intensity to ∼90% of VO_2max_ [199]. Also, in skeletal muscle, HSL is regulated by phosphorylation and by allosteric mechanisms [158,159,200,201].

### 4.3. Intracellular Hormone-Sensitive Lipase Regulation

Muscle HSL activity, stimulated through contractile-based mechanisms [158,202], occurs via a calcium-dependent protein kinase C (PKC) [158,203], which stimulates ERK1/2 to ultimately phosphorylate HSL on Ser^600^ [204]. Already after 1 min of exercise at 30 and 65% of VO_2max_, HSL activity and ERK1/2 phosphorylation increase in moderately trained men without change in blood epinephrine concentration; therefore, HSL activity increases by contraction-based mechanisms only [202]. Instead, in skeletal muscle following 1 min of exercise at both 65% and 90% of VO_2max_, epinephrine concentration increased, and the β-adrenergic pathway resulted activated. The subsequent PKA activation increased HSL Ser^660^ phosphorylation and HSL activity, without a change in Ser^563^ phosphorylation. In conclusion, high-intensity exercise increases adenylate cyclase activity due to β-adrenergic stimulation [205]. Despite that in skeletal muscle catecholamines phosphorylate HSL through PKA, during exercise, the phosphorylation of Ser^563^ on HSL does not increase, even if a several fold increase in epinephrine concentrations is observed, suggesting that, during exercise, HSL Ser^563^ is not a PKA target [144].

Furthermore, AMPK is a major regulator of HSL activity able to override phosphorylation by PKA [160,195]. AMPK exists as a heterotrimeric complex with a catalytic (α) and two regulatory subunits (β and γ) [206] and muscle cells mainly express AMPK complexes containing the α2 catalytic subunit [207,208]. AMPK-α2 isoform is activated during exercise [144,209,210,211,212,213], and such activation is inversely related to glycogen content in skeletal muscle [195,210,213]. Roepstorff et al. [144] investigated the effect of AMPK on HSL activity and Ser^565^ phosphorylation (the presumed AMPK target site) in human skeletal muscle. In moderately trained men during exercise (cycling at 65% VO_2max_), α2AMPK activity was higher in muscles with low glycogen content than in those with high glycogen content. In addition, in human skeletal muscle with reduced muscle glycogen, AMPK phosphorylates HSL on Ser^565^, increasing its activity by 117% at 30 min of exercise. In contrast, in another study, AMPK activation inhibited HSL activity during exercise, although no effect on muscle triacylglycerol breakdown was reported [190]. In addition, HSL translocation to the lipid droplets has been demonstrated in rat skeletal muscle during contractions [214]. In several human studies, dissociations between in vitro HSL activity and net change in IMTG content during exercise have been observed, as increased HSL activity was not always accompanied by a decrease in IMTG [144,179,190].

### 4.4. Adipose Triglyceride Lipase Functions

Adipose triglyceride lipase, also named desnutrin and calcium-independent phospholipase A2ζ (iPLA2ζ)) [215,216,217], is exclusively expressed in type I (oxidative) muscle fibers, suggesting a pivotal role in intramuscular fatty acid handling, lipid storage and breakdown [218]. The functional importance of ATGL is demonstrated by an increased TG hydrolase activity and decreased TG content in myotubes overexpressing ATGL [219]. In addition, mutations of the human genes CGI–58 (also named α/β–hydrolase domain-containing 5, ABHD5), an ATGL activating protein, have been found in patients with neutral lipid storage disease with myopathy, which is characterized by TG accumulation in various tissues, including skeletal muscle [220,221,222,223], suggesting a defect of ATGL function. In skeletal muscle, ATGL content is upregulated 2.5-fold by regular endurance exercise training [196], suggesting a transcriptional increase in its expression. Nielsen et al. [224] found increased ATGL and decreased protein and mRNA content of the ATGL inhibitor G0S2, suggesting increased ATGL activity during fasting, but not after short-term exercise. Ogasawara et al. [225] demonstrated that endurance exercise training increases ATGL protein expression in adipocytes and decreases levels of plasma insulin. The study also demonstrates that exercise training increases mRNA expression of PPAR-gamma coactivator 1alpha (PGC-1), a master regulator of mitochondrial biogenesis, as well as mitochondrial proteins in rat adipose tissue [225].

**Figure 2:**
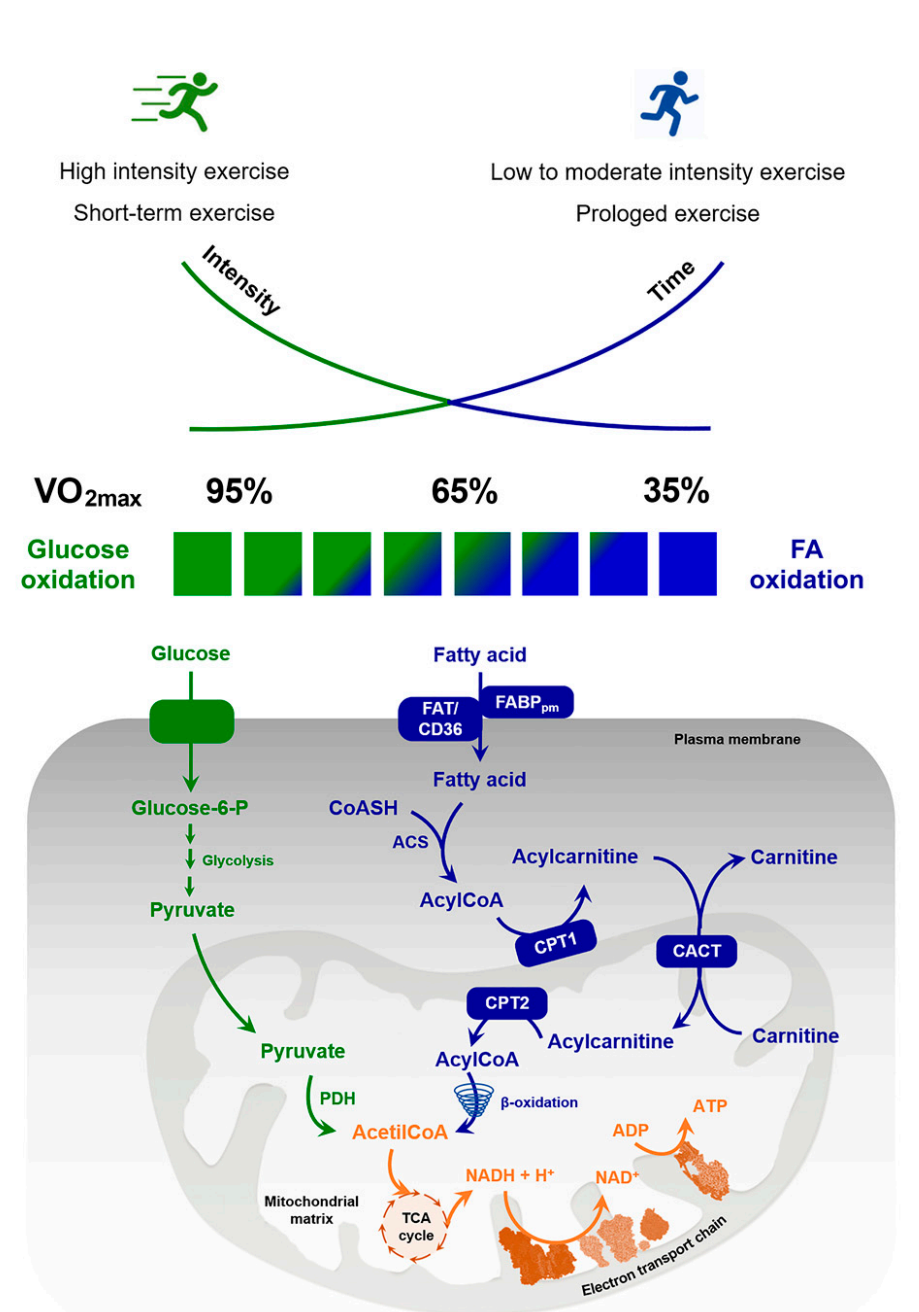
Integration of carbohydrate and lipid metabolism in skeletal muscle cells during exercise. Fat and carbohydrate are important fuels for aerobic exercise and there can be reciprocal shifts depending on the exercise intensity and duration. During prolonged exercise at a low to moderate intensity (35% of VO_2max_), most of the energy requirements for skeletal muscle can be met from predominantly FA oxidation, with a very small contribution from glucose oxidation. Increases in exercise intensity produce a progressive shift in energy contribution from fat towards carbohydrate, until it reaches 95% of VO_2max_, when glucose becomes the main energy source of fuel for skeletal muscle contraction. The figure also shows the regulation involving many sites of control (transport of FFAs into the muscle cell by FA binding protein of the plasma membrane (FABPpm) and FAT/CD36, and into the mitochondria via carnitine palmitoyl transferase (CPT1/CPT2) and the role of carnitine-acylcarnitine translocase (CACT)). When glycolytic flux is increased, as during high-intensity aerobic exercise, the enhanced pyruvate production leads to acetyl-CoA excess and ATP resynthesis at high-energy requirements. At lower exercise intensities or during prolonged exercise, a lower glycolytic rate decreases the supply of glycolysis-derived acetyl-CoA and the reduced sequestration of carnitine enables increased FA import through CPT1 and carnitine shuttle system, favoring utilization of β-oxidation-derived acetyl-CoA in the TCA cycle and ultimately FA oxidation.

Since AMPK in myotubes does not affect ATGL phosphorylation at Ser^406^, differently from adipocytes, AMPK is not an upstream kinase of ATGL in skeletal muscle [226] during submaximal exercise. The lipid-droplet-associated perilipins (PLINs) are also part of the lipolytic machinery in muscle and it has been shown that Perilipin-3 (PLIN3) and PLIN5 interact with ATGL and HSL [227,228]. Figure 2 shows carbohydrate and lipid metabolism shift in skeletal muscle cells depending on exercise intensity and duration.

## 5. Conclusions

Lipids are considered an important source of energy during exercise, especially during low- and moderate-intensity exercise. Exercise-induced FA oxidation is influenced by adipose tissue and IMTG lipolysis, delivery of FA to the exercising muscle, regulation of FA transmembrane transport in muscle cells and mitochondrial metabolism. The regulation of lipid metabolism is an intricate operation. During prolonged exercise, adipose tissue and intramuscular lipolysis are regulated by both contraction and hormonal mechanisms. The major endocrine mechanism is represented by epinephrine acting through HSL and perilipin 1 activation. Muscle HSL activity is stimulated through contractile-based mechanisms. In addition, as an adaptive response during endurance training, the activities of β-oxidation enzymes, the tricarboxylic acid cycle and the electron transport system increase. In this condition, the FA transport through the mitochondrial membrane by carnitine transferase also increases. It seems that the main candidate for FA oxidation regulation is the muscle carnitine content. At high-intensity exercise, the rapid glycolysis provides the mitochondria with excess acetyl-CoA, which is buffered by free carnitine to form acetylcarnitine. Accordingly, a fall in muscle concentration of free carnitine may reduce CPT-1 activity, and thus the ability to transport FAs into the mitochondria, and therefore, also the rate of FA oxidation.

Nonetheless, the mechanisms for increasing lipid metabolism are still to be fully understood as there are many functional and structural steps as fatty acids are mobilized, transported and oxidized in working muscle. For these reasons, it is very important to continue studying the acute and chronic response to the physical activity.

## Figures and Tables

**Table 1:** Articles included in the review.

References	Type of Exercise	Type of Study	Study Sample	Results
Low-Intensity Exercise				
Klein et al. (1994) [63]	4 h of treadmill exercise eliciting an oxygen uptake of 20 mL/kg/min.	Glycerol and free fatty acid rate of appearance and lipid oxidation were evaluated during basal resting conditions and after 4 h of treadmill exercise and 1 h of recovery.	*n* = 5 endurance-trained men; *n* = 5 untrained men.	After 4 h of exercise, the average glycerol and free fatty acid values, was similar in both trained and untrained subjects; but during recovery, glycerol and free fatty acid values decreased more rapidly in trained than in untrained subjects. Triglyceride oxidation was greater during exercise in the trained than in the untrained group.
Wolfe et al. (1990) [70]	4 h of treadmill exercise at 40% maximum O_2_ consumption, and 2 h of recovery.	Total fat oxidation was quantified by indirect calorimetry in response to exercise and in recovery from exercise.	*n* = 5 healthy male subjects.	Rate of appearance of glycerol and free fatty acids increased after 30 min and 4 h of exercise. Lipolysis decreased rapidly from the first 20 min to 2 h of recovery.
Verboven et al. (2018) [71]	12-week exercise training	Abdominal subcutaneous adipose tissue (SCAT) extracellular glycerol concentration and blood flow were measured using microdialysis at rest, during low-intensity endurance-type exercise and post-exercise recovery; at the same time, the response to α-/β-adrenoceptor was evaluated.	*n* = 10 healthy lean insulin-sensitive men *n* = 10 obese insulin-sensitive men *n* = 10 obese insulin-resistant men.	Exercise induce an increase of extracellular glycerol in SCAT in obese IS versus lean IS men: this could be the result of a lower blood flow in subcutaneous adipose tissue in obese IS men. Nonetheless, extracellular glycerol was blunted in obese IR versus obese IS men, despite comparable local blood flow after exercise. SCAT extracellular glycerol was reduced by 60% following local α-/β-adrenoceptor blockade in obese IS but not in obese IR men; in the latter, exercise training did not affect non- adrenergically-mediated lipolysis, despite an improved metabolic profile and body composition.
**Moderate-Intensity Exercise**				
Chycki et al. (2019) [22]	Individuals belonging to the three groups were subjected to progressive exercise protocol on a treadmill at 30%, 50% and 70% VO_2max_, separated by 45 s of passive rest.	Venous blood was collected before, during and after exercise to determinate GH, noradrenaline, insulin, cortisol, glucose, FFA and glycerol.	*n* = 18 healthy trained and untrained men (32 ± 5.4 years): 6 obese subjects; 6 athletic subjects; 6 endurance-trained subjects.	Plasma glucose oxidation increased in relation to exercise intensity, especially in the athletic group, while plasma FFA level decreased with different kinetics in the three groups. Plasma GH increased immediately after exercise and remains high in all groups 45 min into recovery compared to rest. Plasma insulin decreased during exercise in all groups, but to a lesser extent in obese subjects.
O’Hearn et al. (2016) [52]	Two experimental trials conducted in the following way: 90 min baseline period in ambient temperature, followed by 120 min at rest and 30 min exercise at 50% VO_2max_ at either 42 °C or 23 °C.	Metabolic data, heart rate, thermal responses and ventilation were measured throughout the baseline, passive periods and exercise period. Metabolic and ventilation measurements were recorded every 30 min. Blood samples were collected at baseline and 60 and 120 min of the passive period to determine changes in non-esterified fatty acid, TG, phospholipid and TC concentrations.	*n* = 8 healthy males (23–27 years).	Lipid oxidation rates were not different between heat (42 °C) and control (23 °C) conditions, as well as TG, phospholipid and TC levels. However, non-esterified fatty acid concentrations were significantly higher following passive heating (618 µM, 95% CI: 479–757) compared to control condition (391 µM, 95% CI: 270–511), and also following exercise (2036 µM, 95% CI: 1604–2469 for HEAT and 1351 µM, 95% CI: 1002–1699). * CI = confidence interval
	Four experimental trials that consist in a baseline period of 15-min (25 °C) and 60 min of exercise (walking at 50% VO_2max_ in 0 °C; walking at 50% VO_2max_ in 22 °C; running at 70% VO_2max_ in 0 °C and running at 70% VO_2max_ in 22 °C.	Thermal, cardiovascular and oxidative responses were measured every 15 min during exercise. Blood samples for serum non-esterified fatty acids, glycerol, glucose, beta-hydroxybutyrate, plasma catecholamines and serum lipids were collected immediately prior, and at 30 and 60 min of exercise.	*n* = 10 moderately active males (24.3 ± 3.0 years).	During submaximal walking and running, a rise in fat utilization in the cold was seen through lower respiratory quotient (RQ) (−0.03 ± 0.02), greater fat oxidation (+0.14 ± 0.13 g·min^−1^) and contribution of fat to total energy expenditure (+1.62 ± 1.99 kcal·min^−1^). However, serum non-esterified fatty acids, glycerol or catecholamine concentrations did not increase.
Petibois et al. (2002) [69]	A 10 Km run at the individual marathon velocity.	Blood triglycerides and glycerol and other biochemical parameters concentration, during exercise were analyzed.	*n* = 14 marathon runners (28–40 years)	Longer and/or less unsaturated blood fatty acids, a plasma triglyceride decrease, and a glycerol concentration increase were measured in the best runners
Nieman et al. (2017) [72]	Subjects ran on treadmills to exhaustion, with the speed set at 70% of VO_2max_.	Blood samples were collected before and after running to evaluate three cytokines, MCP-1, IL-6 and IL-8, and the stress hormones cortisol and epinephrine. Glycogen concentration was measured in vastus lateralis muscle biopsy. To study lipid metabolic profile was used three independent platforms: ultra-high performance liquid chromatography tandem mass spectrometry optimized for acidic or basic species and gas chromatography–mass spectrometry	*n* = 24 male runners (36.5 ± 1.8 years)	After running, muscle glycogen decreased (33.7% ± 4.2%), while MCP-1, IL-6 and IL-8 increased (1.4 ± 0.1-, 39.0 ± 8.8-, 2.4 ± 0.3-fold, respectively), such as cortisol and epinephrine (95.0% ± 18.9%, 158% ± 20.6%).The metabolomics analysis revealed changes in 209 metabolites, mostly long- and medium-chain fatty acids, fatty acid oxidation products (dicarboxylate and monohydroxy fatty acids, acylcarnitines) and ketone bodies. In this study, the relationship between IL-6 cytokine and adipose tissue lipolysis stimulation was not found.
Nieman et al. (2013) [73]	Subjects ran for 2.5 h/day on treadmills at ∼70% VO_2max_, for 3 days in a row.	75 metabolites, pre-exercise, immediately and 14 h post-exercise, were identified.	*n* = 15 long distance runners (7 males, 8 females; 19−45 years).	Of a total of 75 metabolites, increased immediately following the 3-day running period, 22 were related to lipid and carnitine metabolism, 13 to amino acid and peptide metabolism, 4 to hemoglobin and porphyrin metabolism and 3 to Krebs cycle intermediates (succinate, fumarate, and malate).
Laaksonen et al. (2018) [74]	Participants were divided into efficient (EF) and inefficient (IE) groups based on their mechanical efficiency at 45% of VO_2_ peak intensity during submaximal bicycle ergometer test.	During exercise, muscle blood flow, uptakes of oxygen, fatty acids and glucose were measured using positron emission tomography.	*n* = 17 healthy physically active male (EF: 24 ± 2 years; IE: 23 ± 2 years).	The use of blood glucose and intramuscular FA and glucose appeared to be similar between the two groups. However, EF group had increased muscle FA compared to IE group during exercise which led to higher usage of plasma FA, leading to think that use of plasma FA is important for mechanical efficiency during exercise.
Shaw et al. (2020) [75]	The endurance trained men had regularly competed in cycling and/or triathlon events within the last year, whereas the untrained group were physically active but did not complete regular endurance-type training.	Maximal fat oxidation and maximal oxygen uptake of the two groups was evaluated after exercise test on a cycle ergometer until exhaustion. Blood samples and biopsy were collected to assessed muscle fiber type and proteins involved in intramuscular lipids utilization by immunofluorescence microscopy and immunoblotting.	*n* = 7 endurance trained young males *n* = 8 untrained young males	Endurance-trained subjects displayed a higher maximal fat oxidation rate, a greater proportion of type I muscle fibers and higher intramuscular lipids content compared to untrained individuals. ATGL, HSL, PLIN 2, PLIN 5 and HAD content was ~2–3-fold higher in type muscle fibers compared to type IIa fibers. Consequently, these last were higher in endurance trained individuals.
Dandanell et al. (2018) [76]	A graded exercise test was performed	Plasma maximal rates of fat oxidation and VO_2max_ were determined. Skeletal muscle biopsies were obtained to determine fatty acid oxidation and mitochondrial volume density.	*n* = 8 endurance-trained male cross-country skiers (20–22 years; VO_2max_ 71 mL/min/kg); *n* = 8 healthy untrained male controls (23–24 years; VO_2max_ 48 mL/min/kg).	VO_2max_, plasma maximal rate of fat oxidation, fatty acid oxidation and mitochondrial volume density were higher in the endurance-trained subjects compared to untrained subjects. The mitochondrial volume density, together with central adaptations as VO_2max_, determined the maximal rate of fat oxidation in endurance-trained subjects. Intrinsic mitochondrial changes were not associated with augmented maximal rate of fat oxidation.
Talanian et al. (2010) [77]	Six weeks of high-intensity interval training.	Biopsies were taken before and following 2 and 6 weeks of training from the vastus lateralis muscle.	*n* = 10 untrained females (22 ± 1 years)	High-intensity interval training increases fatty acid transport protein FAT/CD36 (10%) and FABPpm (48%) content in whole muscle; FABPpm and FAT/CD36 content increase in sarcolemmal (23%) and mitochondrial (51%) membranes in human skeletal muscle, respectively.
Jeppesen at al. (2012) [78]	Eight week aerobic training program at 45–80% VO_2max_, with increasing training frequency during the weeks.	FATP1 and FATP4 protein expression in the vastus lateralis muscle.	*n* = 8 healthy males (30 ± 1 years)	FATP4 protein content is increased (33%), whereas FATP1 protein content is reduced (20%) in skeletal muscle. FATP4 protein expression is related to lipid oxidation during endurance exercise.
Petridou et al. (2017) [79]	Participants cycled for 30 min at a heart rate of 130 to 140 beats per minute.	Subcutaneous adipose tissue was sampled at baseline and 5, 10, 20 and 30 min of exercise to determinate triacylglycerol lipase activity and expression; blood was collected for glycerol, non-esterified fatty acid, glucose, lactate, insulin, and catecholamine determination.	*n* = 16 healthy, sedentary men (20–26 years): lean group (*n* = 7; body mass index BMI ≤ 25 kg/m2; body fat < 15%) and an obese group (*n* = 9; BMI > 30 kg/m^2^; body fat > 20%).	Triacylglycerol lipase activity increased at 10 min of exercise in the lean men and returned to baseline at 20 and 30 min; in the obese men, it was higher than baseline at 10, 20 and 30 min and higher than the corresponding values in the lean men at 20 and 30 min. mRNA levels did not change during exercise, but the obese men had lower mRNA levels of ATGL, HSL and CGI-58 compared with the lean men.
**High-Intensity Exercise**				
Romijin et al., (1993) [20]	Different exercise intensity was performed (25%, 65% or 85% of VO_2max_).	Plasma glucose tissue uptake and muscle glycogen oxidation were measured during the different exercise intensities.	*n* = 5 endurance-trained cyclists (24 ± 2 years; VO_2max_ 67 ± 3 mL/min/kg)	Plasma glucose tissue uptake and muscle glycogen oxidation increased in relation to exercise intensity. During at higher intensities exercise, muscle triglyceride lipolysis was stimulated only whereas muscle glycogen and triglyceride oxidation decreased. During recovery from high-intensity exercise, the rate of lipolysis and release of fatty acids into plasma decreased.
Emed et al. (2016) [80]	The 24 h ultramarathon race was performed on an outdoor 400 m athletics track.	Total cholesterol, HDL, triglycerides, ApoB and ApoA1, before and after 400 m run, were assessed.	*n* = 14 male athletes (>18 years old).	No significant modifications in high-density lipoprotein, LDL and ApoA1 levels were measured. A reduction in ApoB levels correlated directly to the distance covered, and an increase in the LDL/ApoB ratio was observed. Lipid profile levels and oxidation of LDL were not acutely altered by prolonged physical activity.
Arakawa et al. (2016) [81]	2-day, 130 km ultramarathon.	Free fatty acids levels, after 1, 3 and 5/6 days 130 km ultramarathon were measured.	*n* = 18 runners (52.1 ± 12.1 years; BMI 21.1 ± 1.6 kg/m^2^).	Free fatty acids levels significantly enhanced during the race periods and stayed elevated after the race. Triglycerides declined on day 2 and day 3, and then returned to baseline level. HDL-C elevated on day 2 and remained elevated up to day 5. T-Chol concentrations decreased on day 2 and day 3, and afterward returned to baseline level.
Yanai et al. (2007) [82]	Participants were subjected to an incremental work test: 3 min of pedaling on a 15 W-loaded cycle ergometer increased by 15 W every minute.	Ventilatory threshold and serum FA changes were evaluated in all participants during exercise; blood samples were obtained at rest, peak work rate and 15 min after exercise.	*n* = 34 healthy female students (20.0 ± 1.0 years; BMI 20.6 ± 1.9): normal participants (*n* = 22) and participants with CD36 deficiency (*n* = 12).	Subjects with CD36 deficiency showed significantly lower ventilatory threshold than normal participants that was related to percentage changes in FA at peak work rate. In normal participants, serum FA levels decreased at peak work rate; in participants with CD36 deficiency, FA levels were not decreased at peak work rate and remained at significantly higher levels than normal participants 15 min after exercise.

* as a confidence interval.
